# miR-342 is associated with estrogen receptor-α expression and response to tamoxifen in breast cancer

**DOI:** 10.3892/etm.2013.915

**Published:** 2013-01-22

**Authors:** YUE-JUN HE, JIAN-ZHONG WU, MING-HUA JI, TAO MA, EN-QI QIAO, RONG MA, JIN-HAI TANG

**Affiliations:** 1 Surgery Department, The Second Affiliated Hospital of Xuzhou Medical College, Xuzhou, Jiangsu 221000;; 2 Center Laboratory; Jiangsu Cancer Hospital, Nanjing, Jiangsu 210000;; 3 Radiotherapy Department, Jiangsu Cancer Hospital, Nanjing, Jiangsu 210000;; 4 Oncology Department of Nanjing Medical University, Nanjing, Jiangsu 210000;; 5 Breast Surgery, Jiangsu Cancer Hospital, Nanjing, Jiangsu 210000, P.R. China

**Keywords:** breast cancer, miR-342, estrogen receptor-α, tamoxifen

## Abstract

Estrogen receptor-α (ERα) is essential for estrogen-dependent growth and its level of expression is a crucial determinant of response to endocrine therapy and prognosis in ERα-positive breast cancer. Breast cancer patients show a wide range of ERα expression levels which change in individual patients during disease progression and in response to systemic therapies. However, little is known concerning how the expression of ERα is regulated in human breast cancer. Recently, several microRNAs (miRNAs) have been identified to regulate ERα expression and to predict ER, progesterone receptor (PR) and human epidermal growth factor 2 (HER2) status. The expression levels of miR-342 and ERα mRNA were analyzed in human breast cancer samples and cell lines by quantitative reverse transcription (RT)-PCR analysis. The correlations between the expression levels of miR-342 and clinicopathological factors were analyzed. Statistically significant associations were observed between miR-342 and ER, HER2 and vascular endothelial growth factor (VEGF) status in the human breast cancer samples and the levels of miR-342 gradually increased as ERα mRNA expression increased. Moreover, ectopic overexpression of miR-342 upregulated the expression levels of the ERα mRNA and significantly sensitized the MCF-7 cells to tamoxifen-induced apoptosis and inhibition of cellular proliferation. These results suggested that miR-342 expression is positively correlated with ERα mRNA expression in human breast cancer and that it may be a significant marker for predicting tamoxifen sensitivity in ERα-positive breast cancer and a potential target for restoring ERα expression and responding to antiestrogen therapy.

## Introduction

Breast cancer is the most common malignancy in females, accounting for 31% of all female cancers. Approximately two-thirds of breast cancers exhibit high concentrations of estrogen receptor (ER). The selective ERα modulator tamoxifen is the most commonly prescribed endocrine therapy. A 5-year treatment of adjuvant tamoxifen therapy has been shown to reduce the 15-year risk for recurrence and mortality in breast cancer patients with ERα-positive cancer (1). However, adjuvant tamoxifen therapy fails in 30–40% of patients and nearly all patients with metastatic disease develop tamoxifen resistance. ERα is essential for estrogen-dependent growth and its level of expression is a crucial determinant of the response to endocrine therapy and the prognosis in ERα-positive breast cancer (2,3). There is no doubt that the more ERα is present in the tumor cells, the greater the likelihood of a favorable response to endocrine therapy (4), but little is known about how the expression of ERα is regulated in human breast cancer.

MicroRNAs (miRNAs) are small (∼21 nucleotides), noncoding RNAs that negatively regulate target genes by predominantly binding to the 3′ untranslated region (3’UTR) of target mRNA, resulting in either mRNA degradation or translational repression (5). Evidence has shown that miRNA mutations or misexpression are associated with various types of human cancer and indicates that miRNAs are able to function as tumor suppressors and oncogenes (6). Previously, studies have shown that microRNA expression profiling also revealed that miRNAs are differently expressed among the molecular subtypes of breast cancer (7,8).

Kondo *et al* reported that miR-206 was markedly decreased in ERα-positive human breast cancer tissues and that the introduction of miR-206 into estrogen-dependent MCF-7 breast cancer cells led to the suppression of ERα expression and growth inhibition (9). Adams *et al* identified that miR-206 decreases endogenous ERα mRNA and protein levels in MCF-7 cells by acting through two specific miR-206 target sites within the 3’UTR of the human ERα transcript (10). Leivonen *et al* previously reported that five ERα-regulating miRNAs, miR-18a, miR-18b, miR-193b, miR-302c and miR-206, directly targeted ERα in 3’UTR reporter assays (11). Furthermore, other studies demonstrated that miR-22 (12,13) and miR-221/222 (14,15) also directly interacted with the 3’UTR region of ERα and regulated ERα expression. Thus, studies have shown critical interactions between ERα and miRNAs and suggested that several miRNAs regulate ERα expression directly or indirectly. It has been shown that the downregulation of miR-342 is associated with ERα-negative breast cancer (8) and tamoxifen-resistant breast tumors (16).

The present study was undertaken to assess the expression of miR-342 and ERα mRNA in human breast cancer samples. Correlations between the expression levels of miR-342 and clinicopathological factors were analyzed. For the first time miR-342 expression was identified as positively correlated with ERα mRNA expression. The ectopic expression of miR-342 upregulated ERα mRNA levels and promoted tamoxifen sensitivity in MCF-7 cells, whereas the knockdown of miR-342 reduced ERα mRNA expression and weakened tamoxifen sensitivity. These results indicated that miR-342 may emerge as a significant marker for the tamoxifen response, as well as as a potential therapeutic target.

## Materials and methods

### Breast cancer tissues and immunohistochemical analysis

A total of 48 breast cancer cases and 24 normal adjacent tissues from female patients with invasive breast carcinoma, who were treated in the Jiangsu Province Cancer Hospital of China between 2010 and 2012, were included in the present study. The study protocol was approved by the institutional review board and conformed to the guidelines of the 1975 Declaration of Helsinki. All patients had undergone surgical treatment for primary breast cancer (either mastectomy or lumpectomy), without previous chemoradiotherapy and were aged between 31 and 82 years old, with a median age of 48. The ERα, progesterone receptor (PR), human epidermal growth factor receptor 2 (HER2) and vascular endothelial growth factor (VEGF) expression status was confirmed by immunohistochemistry (IHC) as follows. One 4-μm section of each submitted paraffin block was first stained with H&E to verify that an adequate number of invasive carcinoma cells were present and that the fixation quality was adequate for IHC analysis. Serial sections (4 μm) were prepared from selected blocks and float mounted onto adhesive-coated glass slides, for staining with monoclonal rabbit anti-human antibodies (Dako, Carpinteria, CA, USA) at a 1:100 dilution. Any brown staining in the invasive breast epithelium was considered a positive result. According to the estimated proportion of tumor cells stained positive, the ER, PR, HER2 and VEGF status was evaluated as follows: Negative (<10%), + (10–30%), ++ (31–50%) and +++ (>50%). HER2 gene amplification was analyzed by fluorescence *in situ* hybridization (FISH) when HER2 status + or ++, the method has been published elsewhere (17).

### Quantitative reverse transcription (RT)-PCR detection of miRNA

Total RNA was extracted from ∼500 mg of frozen breast cancer tissue or ∼1×10^6^ breast cancer cells (MCF-7, SKBR-3,MB-231) using TRIzol reagent (Invitrogen Life Technologies, Carlsbad, CA, USA) according to the manufacturer’s instructions. cDNA was reverse transcribed from the total RNA samples using specific miRNA primers from the TaqMan MicroRNA Assays and reagents from the TaqMan MicroRNA Reverse Transcription kit (Applied Biosystems, Carlsbad, CA, USA). The resulting cDNA was amplified by PCR using TaqMan MicroRNA Assay primers with the TaqMan Universal PCR Master Mix and analyzed with a 7500 ABI PRISM Sequence Detector System according to the manufacturer’s instructions (Applied Biosystems). The relative levels of miRNA expression were calculated from the relevant signals by normalization with the signal for U6 miRNA expression. The assay names for miR-342 were hsa-miR-342-3p (Applied Biosystems).

### Quantitative RT-PCR detection of mRNA

The total RNA (1 μg) was subjected to reverse transcription with random primers in a 20-μl reaction volume using PrimeScript^®^ RT Master Mix (Applied Takara, Dalian, China). The ERα mRNA expression was measured by quantitative RT-PCR with SYBR Premix Ex Taq™ (Applied Takara) and primers for ERα (forward, 5′-TGCCCTACTACCTGGAGAAC-3′ and reverse, 5′-CCATAGCCATACTTCCCTTGTC-3′), using a 7300 ABI PRISM Sequence Detector System according to the manufacturer’s instructions (Applied Biosystems). The relative expression level compared with that of β-actin was calculated using the comparative Ct method.

### Cell culture and transfections

MCF-7 cells (American Type Culture Collection, Manassas, VA, USA) were grown in DMEM (Gibco, Carlsbad, CA, USA) containing 10% fetal bovine serum (FBS) and 2 mM/l L-glutamine and penicillin-streptomycin (50 IU/ml and 50 mg/ml, respectively) at 37°C with 5% CO_2_. The transfection was performed with Lipofectamine™ 2000 Reagent (Invitrogen Life Technologies) according to the manufacturer’s instructions. The miR-342-3p mimics, miR-342-3p inhibitor and the negative control (NC) were purchased from Jima Co., Shanghai, China. The concentration of the mimics and inhibitors were 10 and 20 nM, respectively. The efficiency of the miR-342 transfection was measured by real-time PCR.

### Cell proliferation assay

Following transfection, the MCF-7 cells (5,000 cells per well) were plated in 96-well plates and treated with 10 nM 17β-estradiol (E2, Sigma, St. Louis, MO, USA) alone or in combination with 20 μM tamoxifen (Sigma) for 72 h subsequent to overnight serum starvation. Cell proliferation was documented using a cell counting kit-8 (CCK-8) assay kit (Dojindo Laboratories, Kumamoto, Japan) and recording absorbance at 450 nm with a 96-well plate reader.

### Apoptosis test

Following transfection, the MCF-7 cells (1.5×10^5^ cells per well) were treated with 15 μM tamoxifen for 48 h and then stained with FITC-conjugated anti-Annexin V antibodies. The Annexin V-FITC Apoptosis Detection kit (BD Pharmingen, San Diego, CA, USA) was used to analyze cell apoptosis with flow cytometry (BD Aria; BD Biosciences, Franklin Lakes, NJ, USA).

### Statistical analysis

All statistical analyses were performed using SPSS 17.0. All data are expressed as the mean ± SD of at least 3 independent experiments. The differences between the groups were analyzed using the Student’s t-test or ANOVA; P<0.05 was considered to indicate statistically significant results.

## Results

### Correlations between the expression levels of miR-342 and the clinicopathological factors

The expression levels of miR-342 in the 48 human breast cancer tissues were examined. Quantitative RT-PCR detection analysis showed that the expression levels of miR-342 were markedly higher in the ERα-positive tumors (1.386±0.480) than in the ERα-negative tumors (0.785±0.315; P= 0.000), that the miR-342 expression levels were increased in the HER2-negative tumors (1.416±0.432) compared with the HER2-positive tumors (1.017±0.492; P= 0.001) and that miR-342 expression was upregulated in the VEGF-negative tumors (1.416±0.432) compared with the VEGF-positive tumors (1.088±0.528; P= 0.031). There was no evident relevance between the levels of miR-342 expression and PR, lymph node metastasis status or the pathological grade (P>0.05; Table I). No discrepancy exists in the miR-342 expression between the cancer (1.404±0.529) and cancer adjacent (1.151±0.387; P=0.065) in this study.

### miR-342 expression is positively correlated with ERα mRNA expression in human breast cancer and cell lines

**Figure 1.**
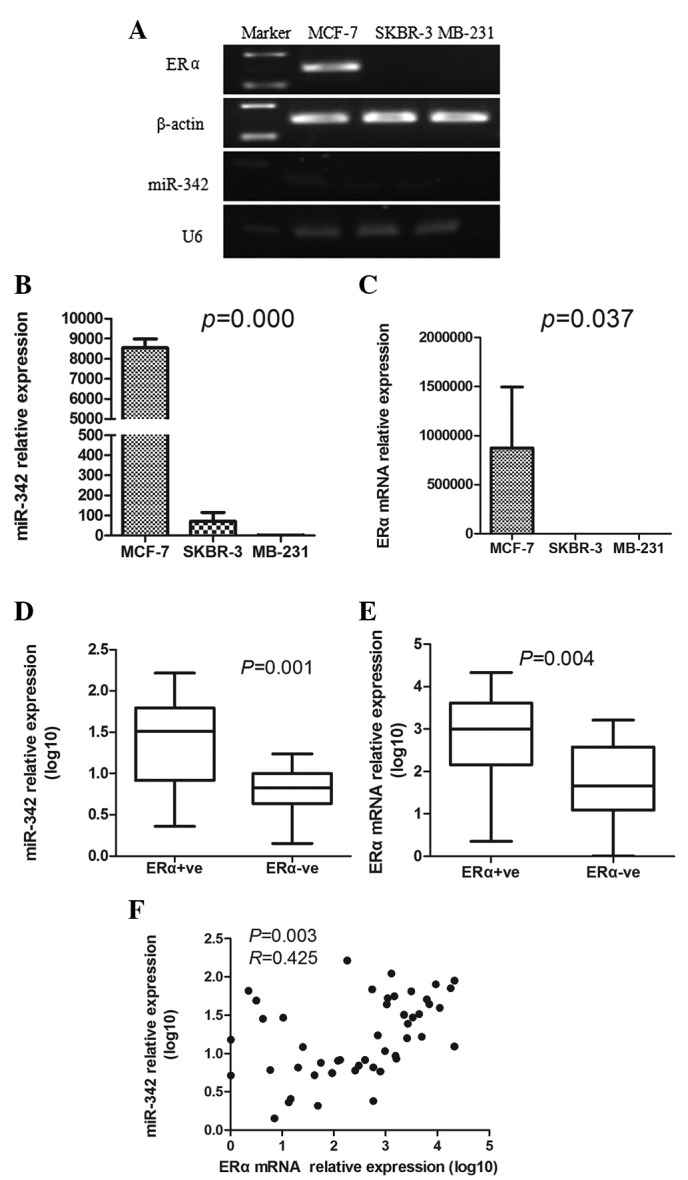
miR-342 is positively correlated with the expression of ERα mRNA in human breast cancer tissues. (A) 3% agarose electrophoresis of PCR products. Quantitative RT-PCR detection analysis showing that expression levels of (B) miR-342 and (C) ERα mRNA are markedly higher in the ERα-positive breast cancer cells (MCF-7) than in the ERα-negative cells (SKBR-3 and MB-231). (D) miR-342 and (E) ERα mRNA increased more in the ERα-positive tumors than in the ERα-negative tumors. (F) Scatterplot shows positive correlation between miR-342 and ERα mRNA expression in the breast cancer tissues. ERα, estrogen receptor α; mRNA, microRNA; RT-PCR, reverse transcription PCR.

First the expression levels of ERα mRNA and miR-342 were assessed in the breast cancer cell lines and the results showed that they were greatly increased in the ERα-positive cells (MCF-7) compared with those in the ERα-negative cells (SKBR-3 and MB-231; P<0.05; Fig. 1A–C). Next the ERα mRNA and miR-342 expression levels were examined in the human breast cancer tissues. As expected, the expression levels of ERα mRNA were much higher in the ERα-positive tumors than in the ERα-negative tumors (2.74±1.14 vs. 1.68±1.02; P= 0.004; Fig. 1E). To analyze the association between the miR-342 expression and the ERα mRNA expression, the expression levels were plotted. The scatterplots showed that miR-342 expression was positively correlated with ERα mRNA expression in human breast cancer (P=0.003; Fig. 1F).

### miR-342 elevates ERα mRNA expression of MCF-7 cells and promotes tamoxifen sensitivity

**Figure 2.**
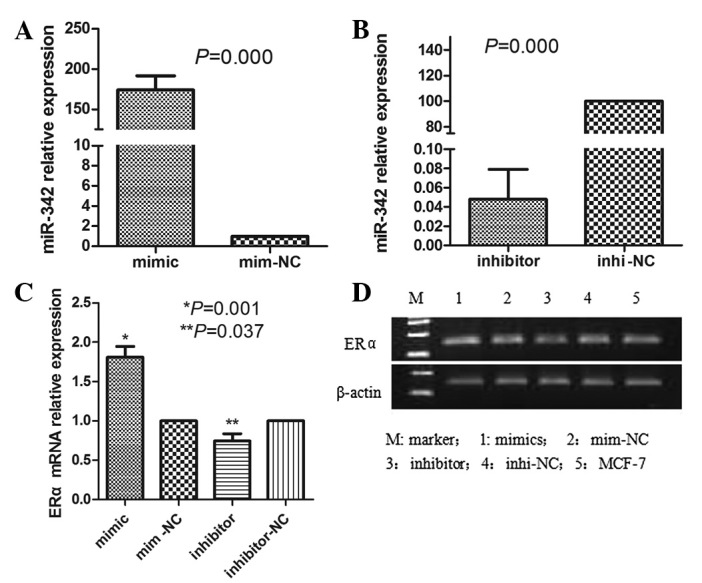
Transfection of miR-342 mimics or inhibitors into estrogen-dependent MCF-7 breast cancer cells changes ERα expression. MCF-7 cells were transfected with either miR-342 mimics (10 nmol/l), inhibitors (20 nmol/l) or the negative control (NC) and incubated for 48 h in a medium containing 10% FBS. The miR-342 levels and ERα mRNA levels were measured by quantitative RT-PCR. (A) miR-342 expression is markedly higher in the cells with the transfection of the miR-342 mimics and lower in the cells with (B) the transfection of the miR-342 inhibitors compared with that of the NC. (C) Quantitative RT-PCR detection analysis and (D) 3% agarose electrophoresis of PCR products showing that ERα mRNA increased in cells with transfection of the miR-342 mimics and decreased in cells with transfection of the miR-342 inhibitors compared with that of the NC. FBS, fetal bovine serum; miRNA, microRNA; ERα, estrogen receptor α; RT-PCR, reverse transcription PCR; mim-NC, mimic NC; inhi-NC, inhibitor NC.

The MCF-7 cells were transfected with the miR-342 mimics at a concentration of 10 nM or with the miR-342 inhibitors at a concentration of 20 nM. The control groups were transfected with the miR-342 NCs or with the miR-342 inhibitor NCs. To examine the efficiency of the transfection, total RNA was extracted and the miR-342 level was measured by real-time PCR 48 h post-transfection. The results showed that the miR-342 expression was significantly increased in the MCF-7 cells following transfection with the miR-342 mimics, when compared with control group treated with the mimic NCs (P=0.000; Fig. 2A). The miR-342 expression was markedly lower when using the miR-342 inhibitors than when using the miR-342 inhibitor NCs (P=0.000; Fig. 2B). The ERα mRNA expression was analyzed by RT-PCR, which showed that the levels of ERα mRNA expression were upregulated in the group transfected with the miR-342 mimics compared with those in the control group and decreased in the group transfected with the miR-342 inhibitors compared with those in the control group (Fig. 2C and D).

**Figure 3.**
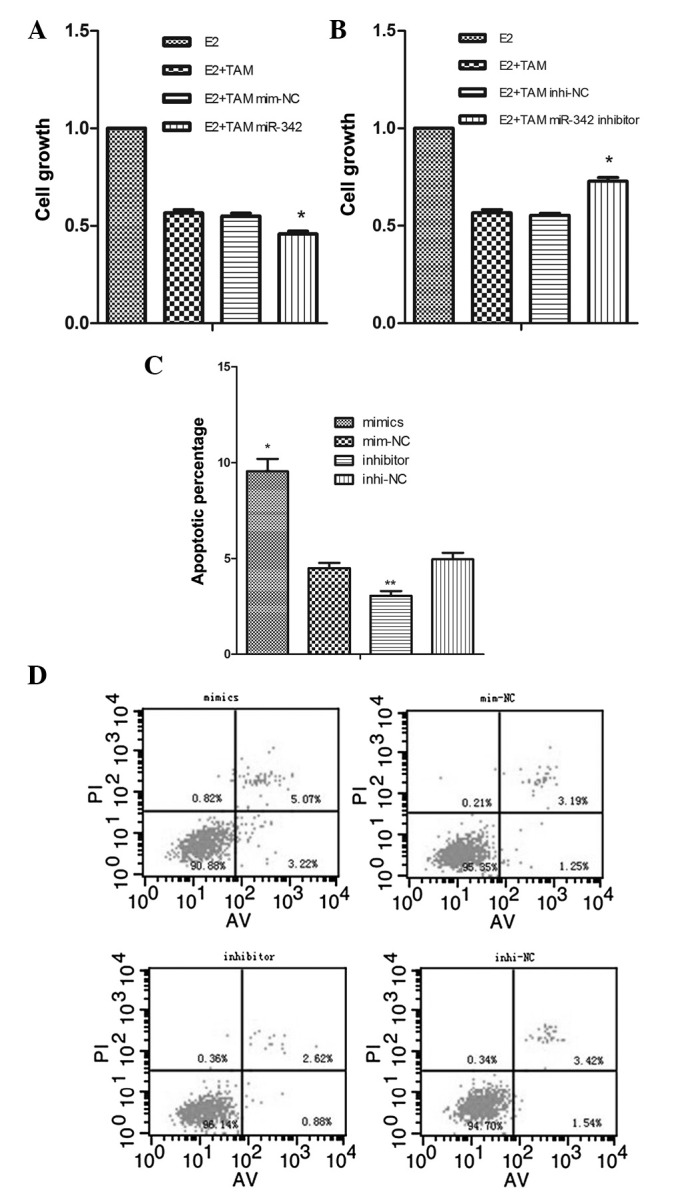
Transfection of miR-342 into MCF-7 cells promotes tamoxifen sensitivity. MCF-7 cells were transfected with either (A) miR-342 mimics (10 nmol/l), (B) inhibitors (20 nmol/l) or the negative control (NC) and treated the next day with 10 nM E2 alone or in combination with 20 μM tamoxifen for 72 h. Cell growth was measured by a CCK-8-based cell proliferation assay. Data are presented as mean ± SE of three independent experiments relative to E2 treated MCF-7 cells. Compared with the NC group, (A) ^*^P= 0.001; and (B) ^*^P=0.000. (C and D) MCF-7 cells were transfected and treated the next day with 15 μM tamoxifen for 48 h and the apoptosis was quantitated with flow cytometry. (C) Compared with the NC group, ^*^P= 0.002; ^**^P= 0.011. mim-NC, mimic NC; inhi-NC, inhibitor NC, TAM, tamoxifen, miR-342, microRNA-342.

As miR-342 is not differently expressed between the breast cancer and cancer adjacent tissues, we forecast that miR-342 would not play a tumor-suppressive or tumor-promotive role in breast cancer development. To understand the functional role of miR-342, the impact of miR-342 on cellular proliferation was evaluated using CCK-8 in the MCF-7 cells. The results showed that 96 h after the use of miR-342 mimics or inhibition transfection, the overexpression or suppression of miR-342 was not able to change cellular proliferation. Transfection with the miR-342 mimics compared with the NC, (2.460±0.036 vs. 2.517±0.050, respectively; P=0.188). Transfection with the miR-342 inhibitors compared with the NC, (2.363±0.1999 vs. 2.547±0.080, respectively; P=0.212). However, in the presence of 20 μM tamoxifen for 72 h, ectopic miR-342 expression was able to suppress cellular proliferation to a greater extent following transfection with the miR-342 mimics than the cells transfected with the NC (0.459±0.013 vs. 0.55±0.015, respectively; P=0.001; Fig. 3A). By contrast, the suppression of miR-342 is able to inhibit cellular proliferation less following the transfection with miR-342 inhibitors than the cells with the NC (0.729±0.019 vs. 0.554±0.01, respectively; P=0.000; Fig. 3B).

As tamoxifen is known to induce apoptosis in breast cancer cells (18), the potential role of miR-342 in promoting tamoxifen-mediated apoptosis was explored. For this purpose, miR-342-overexpressing or miR-342-suppressing MCF-7 cells were treated with 15 μM tamoxifen for 48 h, then cell apoptosis was analyzed with flow cytometry under the same conditions. The results showed that the apoptotic percentage was higher in the miR-342-overexpressing cells than in the NC (9.54±1.14 vs. 4.50±0.46%; P=0.002). Conversely, the apoptotic percentage was lower in the miR-342-suppressing MCF-7 cells than in the NC (3.06±0.42 vs. 4.95±0.59%; P= 0.011; Fig. 3C and D). This series of analyses demonstrated that the miR-342 indeed plays a key role in changing the response of MCF-7 cells to tamoxifen.

## Discussion

The present study demonstrated that the expression of miR-342 in the ERα-positive breast cancer tumors and cells was significantly greater than that in the ERα-negative breast cancer tumors and cells. The study reported for the first time that the levels of miR-342 expression were positively correlated with ERα mRNA expression and also revealed a correlation between increased tamoxifen sensitivity and the elevated levels of ERα mRNA by augmenting the miR-342 expression.

In experimental models, a single miRNA is able to regulate a number of genes (19). It has been reported that miR-22 is downregulated in ERα-positive human breast cancer cell lines and clinical samples (13). miR-22 inhibits estrogen signaling by directly targeting the ERα mRNA (12). miR-221/222 negatively regulates ERα and is associated with tamoxifen resistance in breast cancer (14). Previous studies have shown that miR-342 is an ERα-associated miRNA (8). The results of the present study show that the expression levels of miR-342 were markedly higher in the ERα-positive breast cancer tumors than in the ERα-negative tumors and that the levels of miR-342 gradually increased as ERα mRNA expression increased, suggesting that miR-342 is a key factor for the regulation of ERα expression in the development and progression of human breast cancer.

Endocrine therapy has become the most significant treatment option for women with ERα-positive breast cancer, with ∼70% of primary breast cancers expressing ERα. The selective ERα modulator tamoxifen is the most commonly prescribed endocrine therapy. Currently there are only a few useful tumor markers to guide management decisions for women with ERα-positive breast tumors. Cittelly *et al*(16) demonstrated that miR-342 was markedly suppressed in multiple tamoxifen-resistant breast tumor cell lines and in primary breast tumors of patients whose tamoxifen therapy failed. Significantly, the reintroduction of miR-342 sensitized the refractory breast tumor cells to tamoxifen therapy, suggesting that miR-342 is a significant regulator of the tamoxifen response. In the present study, miR-342 expression was shown to be positively correlated with the expression of ERα in human breast cancer tissues and the introduction of miR-342 into estrogen-dependent breast cancer cells was shown to upregulate ERα expression and enhance tamoxifen sensitivity with decreased cellular proliferation and increased apoptosis. By contrast, inhibition of miR-342 in the MCF-7 cells downregulated the ERα expression and weakened the response to tamoxifen, with increased cellular proliferation and decreased apoptosis. Based on these observations, we propose that the levels of miR-342 expression that correspond to the ERα mRNA expression locus may act as a biomarker for tamoxifen sensitivity in ERα-positive breast cancer.

Cittelly *et al*(16) reported that there was no evident association between the direct targets of miR-342 and the tumor cell response to tamoxifen. Ingenuity Pathway Analysis of the entire set of genes significantly altered by miR-342 revealed a significant association between the miR-342-regulated genes and cell apoptosis. This result is consistent with the observations of the present study that showed that ectopic miR-342 expression sensitized MCF-7 cells to tamoxifen-induced apoptosis. Similarly, miR-342 expression in colorectal cancer cells results in tumor cell apoptosis (20). Nevertheless, the activity of miR-342 appears to differ functionally in colorectal and breast tumor cells. The results of the present study indicated that miR-342 expression alone was not sufficient to induce cell death, but that miR-342 sensitizes cells to cellular proliferation inhibition and apoptosis associated with tamoxifen exposure.

In addition, the results showed that the levels of miR-342 expression increased in VEGF-negative, HER2-negative and Luminal-A breast cancer samples. As the VEGF-negative, HER2-negative and Luminal-A signals indicate a good prognosis, miR-342 may be a biomarker of predicting a good prognosis for breast cancer.

In conclusion, the present data indicated for the first time that miR-342 expression is positively correlated with the expression of ERα mRNA in human breast cancer tissues and that the introduction of miR-342 into estrogen-dependent breast cancer cells enhances tamoxifen sensitivity. miR-342 may be a novel candidate for ERα-specific endocrine therapy in breast cancer.

## Figures and Tables

**Table I.** Correlation between the miR-342 expression level and the clinicopathological characteristics of breast cancer.

		Relative level of miR-342 (log10)	
Variable	n	Mean ± SD	P-value
Age (years)			
≥48	16	1.202±0.575	0.935
<48	32	1.215±0.492	
Pathological grade			
I, II	36	1.243±0.560	0.367
III	12	1.116±0.353	
Lymph node status			
Metastasis	32	1.218±0.533	0.893
No metastasis	16	1.197±0.494	
ER			
Negative	14	0.785±0.315	0.000
Positive	34	1.386±0.480	
PR			
Negative	20	1.042±0.531	0.054
Positive	28	1.332±0.477	
HER2^a^			
Negative	20	1.482±0.423	0.001
Positive	28	1.017±0.492	
VEGF			
Negative	18	1.416±0.432	0.031
Positive	30	1.088±0.528	
Molecular Subtype			
Luminal A (ER^+^, HER2^−^)	16	1.624±0.333	0.000
Luminal B (ER^+^, HER2^+^)	18	1.175±0.499	
HER2 overexpression (ER^−^, HER2^+^)	10	0.732±0.340	
Triple-negative (ER^−^, PR^−^, HER2^−^)	4	0.918±0.223	
AJCC Clinical Stage			
I	12	1.150±0.562	0.553
IIA	30	1.193±0.510	
IIB^b^	6	1.423±0.480	

a HER2-positive: HER2(+++) or Fish(+).

b There were no patients at clinical stage III or IV in the present study. ER, estrogen receptor; PR, progesterone receptor; HER2, human epidermal growth factor receptor 2; VEGF, vascular endothelial growth factor.
